# Integrative multi-omics profiling of mild cognitive impairment in older patients combined with coronary heart disease: a cross-sectional cohort study

**DOI:** 10.3389/fcvm.2026.1602893

**Published:** 2026-02-23

**Authors:** Tong Li, Yueying Zhang, Jiaqi Hui, Wenxin Zou, Zhongwen Qi, Fengqin Xu

**Affiliations:** 1 Beijing University of Traditional Chinese Medicine, Beijing, China; 2 The Second Department of Geriatrics, Xiyuan Hospital, China Academy of Chinese Medical Sciences, Beijing, China; 3 Laboratory of Combining Diseases and Evidence to Prevent Vascular Ageing, National Administration of Traditional Chinese Medicine, Beijing, China; 4 Department of Integrated Traditional and Western Medicine, Peking University Health Science Centre, Beijing, China

**Keywords:** coronaryheartdisease, cross-sectional cohort study, elderlypopulation, mildcognitiveimpairment, multi-omics profiling

## Abstract

**Introduction:**

With the escalating global burden of population ageing, the comorbidity of geriatric coronary heart disease (CHD) and mild cognitive impairment (MCI) has emerged as a significant public health concern. This study aims to elucidate the underlying clinical disease features of MCI in elderly CHD patients through comprehensive multi-omics analyses and to establish a detailed phenomics profile.

**Methods:**

A cross-sectional study design will be implemented, enrolling 364 elderly patients diagnosed with CHD. Participants will be divided into MCI and cognitively normal groups based on Montreal Cognitive Assessment (MoCA) scores. Extensive data collection will include demographic characteristics, clinical parameters, laboratory investigations, and echocardiographic findings. These clinical data will be integrated with multi-omics analyses (including genomics, proteomics, and metabolomics) to identify differential biomarkers between the two groups.

**Discussion:**

This study anticipates constructing an integrative network map delineating the multi-omics mechanisms underlying MCI in elderly CHD patients by correlating macroscopic clinical phenotypes with microscopic molecular characteristics. The findings are expected to provide a scientific foundation for early detection and intervention strategies. While acknowledging the inherent limitations of the cross-sectional design, future prospective studies are warranted to validate these findings. This investigation offers novel insights and potential therapeutic strategies for the prevention and management of comorbid CHD and MCI in the ageing population. This study has completed registration in the International Traditional Medicine Clinical Trial Registration Platform (ITMCTR2024000871).

**Clinical Trial Registration:**

https://itmctr.ccebtcm.org.cn, identifier ITMCTR2024000871.

## 1 Introduction

As the global population ages, age-related diseases, especially cardiovascular and cerebrovascular diseases, have become a heavy burden. The elderly population in China is expected to reach 487 million by 2050 (1), and ageing leads to an increased prevalence of multimorbidity. Mild Cognitive Impairment (MCI) represents a common co-morbidity, with the incidence of cognitive dysfunction in patients with coronary heart disease (CHD) being significantly higher than in the general population (2, 3). MCI not only progresses to dementia but also elevates the risk of cardiovascular events (4). Therefore, active prevention and treatment of combined CHD and MCI in the elderly can not only slow down the transformation of MCI to dementia but also reduce the incidence of cardiovascular events. There is increasing evidence that there is a strong biological link between the heart and the brain, and pathological changes in the coronary arteries can affect the brain through the heart-brain circuit (5). Atherosclerosis-related vascular pathological processes, such as thickening of the carotid intima-media, may play a significant role in cognitive impairment (6). Currently, there is a lack of consensus on the phenotypic profile and prevention and treatment strategies of coronary artery disease combined with MCI in the elderly, and there is an urgent need for multi-omics technologies combined with clinical studies to explore the underlying pathogenesis of this disease. Multi-omics technologies can reveal the mechanisms of complex diseases and personalised treatments by integrating biological data (7), and have been widely used in the study of cardiovascular and cerebrovascular diseases to help demonstrate disease phenotypes more effectively and provide new perspectives for treatment.

## 2 Materials and methods

### 2.1 Study design and participant selection

**Figure 1:**
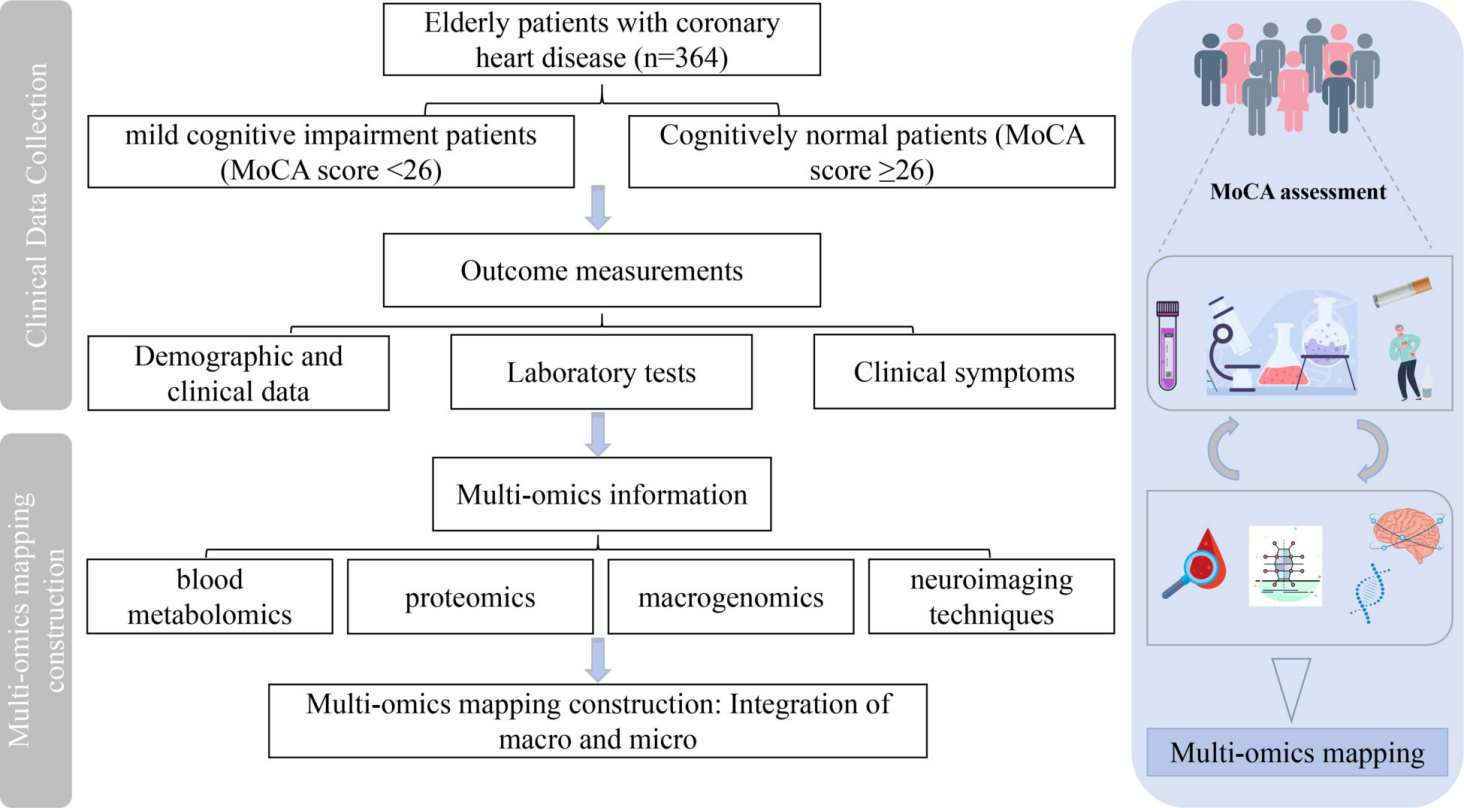
Design and workflow for the construction of a multi-omics mapping of MCI in elderly CHD patients. Vector images by Freepik (https://www.freepik.com/) and Vecteezy (https://www.vecteezy.com/).

A cross-sectional survey will be conducted, enrolling elderly patients with CHD who meet predefined inclusion and exclusion criteria. They will be categorised into the MCI group (observation group) and the normal cognitive function group (control group) based on the Montreal Cognitive Assessment (MoCA) score. The study started in December 2024 and is expected to end in August 2025. This investigation is based on a research project examining the biological essence of vascular senescence in elderly CHD patients with comorbid MCI and the promotion of traditional Chinese medicine applications guided by heart-brain-synthesis treatment principles. The project, sponsored by Xiyuan Hospital of China Academy of Chinese Medical Sciences (CATCM), aims to obtain multi-omics information on elderly patients with CHD and concurrent MCI to construct a comprehensive phenomics mapping (Figure 1). This study has completed registration in the International Traditional Medicine Clinical Trial Registration Platform (ITMCTR2024000871) and was conducted in the outpatient and inpatient departments of Xiyuan Hospital, China Academy of Chinese Medical Sciences. The ethics committee at the Xiyuan Hospital approved the study (Approval ID: 2024XLA216-2). The specific overview of the study procedure is shown in Figure 1.

### 2.2 Recruitment and screening

Eligible patients will be identified according to diagnostic descriptions and the International Classification of Diseases (ICD-10 and ICD-11). Recruitment will be conducted both offline at the outpatient clinics and wards of Xiyuan Hospital, China Academy of Chinese Medical Sciences, and online through advertisements. Participant enrollment is ongoing between December 27, 2024, and August 31, 2025. The diagnostic criteria for coronary artery disease refer to the 2023 AHA/ACC/ACCP/ASPC/NLA/PCNA Guidelines for the Management of Patients with Chronic Coronary Artery Disease: diagnostic criteria for coronary artery disease in the report of the Joint American Heart Association/American College of Cardiology Committee on Clinical Practice Guidelines (7); MCI is identified using a standardized assessment protocol. The diagnostic framework follows the 2018 Chinese Guidelines for the Diagnosis and Treatment of Dementia and Cognitive Impairment (8).

Cognitive assessments will be performed by two trained research staff who have received standardised training in the administration and scoring of the MoCA and will be responsible for determining MCI status in enrolled patients with CAD.

The diagnostic workflow for cognitive impairment includes:

(1) initial cognitive screening using the MoCA;
(2) adjustment of MoCA scores according to educational level (one-point correction for participants with ≤12 years of education);
(3) integration of cognitive assessment results with relevant clinical information;
(4) classification of cognitive status according to guideline-based criteria.

For this study, participants meeting the following criteria will be eligible for enrollment:

(1) Patients in stable condition after admission for Acute Coronary Syndrome (ACS) or after coronary revascularisation (PCI, CABG); with coronary angiography or CTA indicating ≥50% stenosis in at least one coronary artery;
(2) MoCA scale: score <26. The scale is scored out of 30, with ≥26 being normal, 18–25 being MCI, 10–17 being moderate, and <10 being severe. If the subject has ≤12 years of education (high school level), 1 point may be added to the result, up to a total of 30 points.

Final eligibility was determined based on standardised assessment results and predefined inclusion and exclusion criteria. All participants provided written informed consent. The inclusion and exclusion criteria are shown in Table 1.

**Table 1:** Inclusion and exclusion criteria for the study.

Inclusion criteria
Age ≥ 65 and ≤ 80 years, gender is not limited
Visual and auditory discrimination is relatively intact, and the body can move freely and cooperate in completing the relevant assessment.
Agreed to participate in the study, and the patient signed the informed consent form himself/herself
Not participating in other clinical trials.
Exclusion criteria
Dementia of all causes, including AD, VaD, or other types of dementia such as Parkinson's disease, Huntington's disease, normal pressure hydrocephalus, brain tumours, progressive supranuclear palsy, epilepsy, chronic subdural haematomas and multiple sclerosis, assessed by the DSM-IV diagnostic criteria for dementia
History of severe head trauma with persistent neurological deficits or known structural brain abnormalities
Previous mental abnormality, drug or alcohol abuse or dependence within the previous 5 years
Patients with severe hepatic or renal insufficiency, e.g., ALT or AST above 1.5 times the normal value, or above the upper limit of the normal value of serum Cr, coagulation disorders, etc., who are not expected to be able to complete the test
Pacemakers, artificial heart valves, electrodes, various metal foreign bodies retained in the body after surgery or due to trauma; patients whose vital signs are not stable and whose condition is serious (e.g., coma, dementia, etc.) who are unable to communicate with the physician normally, and who are not able to undergo magnetic resonance examination because of the possibility of accidents during the examination process; and subjects who are not able to co-operate with the examination for other reasons.

### 2.3 Sampling and sample size

The sample size is calculated according to the sample size estimation method of binary logistic regression model to calculate the minimum number of cases for logistic regression study [*N* = 10k/p, where p is the probability of occurrence of the smallest event in the population, and k is the number of the main covariates (the number of factors)] (9), and the group's previous study found that (10), the prevalence of MCI in patients with CHD aged over 55 years is 55%. With k = 20 main covariates planned for inclusion, the minimum required sample size is estimated to be 364 participants.

### 2.4 Outcome measurements

#### 2.4.1 Demographic and clinical data

Standardised questionnaires will be used to collect demographic, symptom and medical history data. Sociodemographic information includes age, gender, body mass index(BMI), hours of sleep, marital status, literacy, nature of work and type of occupation. Clinical data includes past and present illnesses (acute and chronic), substance use and family history. Coronary heart disease-related information includes duration of CHD, number of diseased vessels and degree of stenosis, whether PCI was performed, and medication. Past medical history, smoking history, and alcohol consumption history will be also recorded. The vital sign data, such as temperature, respiration, pulse, and blood pressure, will be also included.

#### 2.4.2 Cognitive assessment

At baseline, all participants will undergo a cognitive assessment. Overall cognitive function will be assessed using the MoCA scale. Neuropsychological scale screening tests are of significant value in identifying cognitive impairment. Currently, the Brief Mental State Examination (MMSE) and the MoCA are the most commonly used cognitive assessment tools. Of the two, the MoCA is more comprehensive and sensitive than the MMSE, covering seven dimensions, including attention and concentration, executive function, memory, language, visual-spatial ability, orientation, and abstraction, and is suitable for use in a wide range of clinical practices. In a study of cognitive function assessment in middle-aged and elderly patients (11), the evaluation results of the MoCA scale are more accurate than those of the MMSE, providing more objective results for the assessment of cognitive dysfunction, which is worth promoting and applying in clinical practice. Moreover, MoCA is advantageous in the diagnosis of MCI in the elderly (12, 13). Therefore, the group chose MoCA as a screening tool for determining the presence of MCI.

#### 2.4.3 Laboratory tests

Test results within 7 days before and after screening are acceptable. The laboratory test contents are shown in Table 2.

**Table 2:** The laboratory tests.

Category	Test indicators
Complete blood count	White blood cell count (WBC), Neutrophil count (NEUT), Lymphocyte count (LYMPH), Monocyte count (MONO), Erythrocyte distribution width (RDW-CV)
Liver function	Alanine aminotransferase (ALT), Aspartate aminotransferase (AST), Total bilirubin (TBIL), Total bile acids (TBA), Albumin (ALB)
Kidney function	Uric acid (UA), Urea nitrogen (BUN), Creatinine (Cr), Glomerular filtration rate (GFR)
Lipid profile	Total cholesterol (TC), Triglycerides (TG), Low-density lipoprotein (LDL-C), High-density lipoprotein (HDL-C)
Cardiovascular markers	Troponin (cTnT), N-terminal B-type natriuretic peptide precursor (NT-proBNP)
Coagulation function	Prothrombin time (PT), Activated partial thromboplastin time (APTT), Thrombin time (TT), Fibrinogen concentration (FBg), D-dimer
Glycemic control	Glycated haemoglobin (HbA1c)
Echocardiography	Left ventricular ejection fraction (LVEF), Interventricular septal thickness (IVS), Aortic diameter (AoD), Aortic sinus diameter (Sinus of Valsalva), Ratio of mitral early diastolic flow velocity to mitral annular early diastolic motion velocity (E/e’)

#### 2.4.4 Multi-omics profiling

Multi-omics techniques will be applied in this study to my high-throughput data for differential markers at the micro-level of MCI in older adults with CHD. Early in the morning of the second day, the enrolled patients will have 2 mL of peripheral venous blood drawn by the nurse in the blood collection room in an EDTA anticoagulation tube. The upper layer of plasma will be retained after centrifugation (3,000 rpm, 4 °C for 15 min), and then frozen in a −80 °C refrigerator for non-target metabolomic and proteomic assays. Collect 3–5 g of fresh faecal samples, place them in sterile collection tubes and store them in a −80 °C refrigerator within 30 min for macrogenomics testing. A cranial nuclear magnetic examination will be performed to collect information on neuroimaging techniques. For an overview of all research and design objectives of the Multi-omics, see Table 3.

**Table 3:** Multi-omics studies and design objectives.

Multi-omics profiling	Clinical sample	Design objectives
Untargeted metabolomics	Plasma	Screening for key differential metabolites
Proteomic	Plasma	Screening for key differential proteins
Macrogenomic	Fresh faecal	Analysing changes in the microbiota of MCI in elderly CHD patients
Neuroimaging	Brain MRI scans (T1W1 + Flair+rs-fMRI + 3D-ASL)	Exploring differences in brain structure and function

##### 2.4.4.1 Untargeted metabolomics analysis

The above plasma samples will be pre-separated using an ACQUITY UPLC T3 column, and the metabolic analytes in the pre-separated samples will be analyzed by Xevo G2-XS QToF high-resolution mass spectrometry. The reproducibility of the within-group samples and quality control samples will be assessed using principal component analysis (PCA) and Spearman correlation analysis. The identified metabolites will be annotated by searching against the KEGG, HMDB, and Lipid Maps databases to determine their classification and enriched metabolic pathways. Enrichment analysis and pathway topology analysis will be performed using MetaboAnalyst software to identify key differential metabolic pathways in plasma.

##### 2.4.4.2 Proteomic profiling

The above plasma samples will undergo protein-level quality control. Briefly, proteins will be extracted from the samples, and the protein concentration will be quantified using either the Bradford assay or the BCA assay. The protein samples will then be analyzed by SDS-PAGE to assess sample integrity and quality. Following peptide preparation, the protein samples will undergo reductive alkylation and will be digested with trypsin. The resulting peptide mixtures will be desalted, quantified, and an aliquot will be taken for liquid chromatography–tandem mass spectrometry (LC-MS/MS) using data-independent acquisition (DIA). Raw data will be processed using Spectronaut™ software for protein identification and quantification. The resulting protein abundance data will undergo statistical and bioinformatics analyses to identify potential biomarkers and to explore their associations with cognitive impairment and CHD.

##### 2.4.4.3 Macrogenomic sequencing

Fresh faecal specimens will be collected from participants. A sterile faecal spoon will be used to sample material from at least three distinct sites within each stool specimen, with a target volume of 3–5 g, while avoiding contamination. Samples will be stored at −80 °C within 30 min of collection.Gut microbial DNA will be extracted from the faecal samples, and shotgun metagenomic sequencing will be performed to characterize the composition of the gut microbiota. Gene species will be annotated to identify differences in the relative abundance of the top ten most prevalent gut microbial species.Principal coordinate analysis (PCoA), Anosim analysis, and species LEfSe analysis will be conducted at each taxonomic level to evaluate the association between gut microbiota alterations and cognitive dysfunction in older adults with CHD.

##### 2.4.4.4 Neuroimaging acquisition and analysis

Brain MRI will be conducted on a 3.0 T scanner (GE Discovery MR750). The scan protocol will consist of the following sequences: (1) fluid-attenuated inversion recovery (FLAIR); (2) T1-weighted (T1w) imaging; (3) resting-state functional MRI (rs-fMRI); and (4) three-dimensional arterial spin labeling (3D-ASL). Rs-fMRI and 3D-ASL acquisition parameters will be as follows: (1) rs-fMRI: TR = 2000ms, TE = 30 ms, slice thickness = 3.5 mm, flip angle = 90°, volume = 240, scan time = 6 min; (2) 3D-ASL: TR = 4,632 ms, TE = 10.5 ms, slice thickness = 4 mm, PLD = 1,525 ms, scan time=3min15s.The acquired image data will be preprocessed, and the raw images will be segmented to extract the white matter of each brain region, grey matter volume, cerebral white matter high signal, lacunar infarct foci, histological matrix features, functional connectivity of the whole brain, regional homogeneity, cerebral blood flow, and neurovascular coupling information. Algorithms such as logistic regression, support vector machines, and random forests will be applied to determine the final key differential features.

During the scanning process, participants will be asked to relax, close their eyes, and avoid falling asleep to minimise motion artefacts. To ensure the quality of the images, the mouths of the examined patients will be as free of dentures as possible, and the raw image data will be examined to exclude subjects with visible image artefacts.

#### 2.4.5 Clinical symptom evaluation

Research has shown (14) that cognitive function declines significantly faster in the years following the diagnosis of CHD than before the onset of the disease. Clinical symptoms are often early warning signs of subsequent disease progression. In elderly patients with CHD, some of the clinical symptoms manifested may have a significant impact on whether cognitive impairment occurs. Systematically collecting and analysing patients’ symptom information not only helps to identify potential risk factors at an early stage but also allows for timely treatment adjustments, thus effectively preventing cognitive impairment from occurring. This will not only improve patients’ quality of life but may also delay or even avoid cognitive decline. The symptom information will collect from patients mainly includes: the degree and frequency of chest pain and chest tightness episodes, Canadian Cardiovascular Society (CCS) grading assessment, long-term physical discomfort symptoms, diet, sleep, exercise, and physical appearance and morphology.

#### 2.4.6 Quality assurance and control measures

All researchers completed Good Clinical Practice (GCP) training and received instruction on the study procedures prior to study initiation, including administration of the MoCA scale, and standardized collection of blood and faecal samples, to ensure rigor and consistency in data and sample acquisition. All investigators demonstrated a thorough understanding of the clinical study protocol, were proficient in all study procedures, and were authorized by the Principal Investigator to conduct the study.

#### 2.4.7 Statistical analysis

SPSS 25.0 will be used to analyse the data, and the descriptive statistics will be presented as mean ± standard deviation (x ± s) or as a percentage for each group. Based on the normality of the distribution of continuous variables, either the t-test or the Mann–Whitney *U*-test will be used to detect differences in baseline characteristics. The unordered categorical variables of baseline data between the groups will be tested by the Chi-square test or Fisher's exact test. To identify the influencing factors of MCI combined with CHD in the elderly, clinical data (e.g., past medical history, family history, laboratory tests and clinical symptoms) will be analysed using the aforementioned univariate analysis method. Variables with a *P*-value < 0.1 will be selected and incorporated into subsequent multivariate logistic regression models to control for confounding biases. The predictive value of the relevant indices for the outcome will be assessed using the receiver operating characteristic (ROC) curves.

We will integrate multi-omics features with clinical phenotypic data to construct a multi-omics mechanism network diagram corresponding to the clinical phenotype of MCI combined with CHD in the elderly. We will establish the correlation between micro-phenotypic features (multi-omics data and laboratory tests) and macro-phenotypic clinical manifestations (physiological and pathological characteristics). Before multi-omics integration, feature selection and dimensionality reduction analysis will be performed at each omics level. Principal Component Analysis (PCA) will be used to visualise the data distribution of samples across different groups, and Partial Least Squares Discriminant Analysis (PLSDA) will be employed to observe the differential data distribution among sample groups, screening out metabolites, proteins, microbial features, and imaging phenotypes significantly associated with MCI combined with CHD in the elderly. Functional enrichment analysis, such as GO and KEGG pathway analysis, will be conducted to preliminarily understand the biological significance of each omics data. Spearman correlation will be used to evaluate the co-expression patterns between different types of omics data features and clinical phenotypes in each sample group. Based on the results of correlation analysis, we will apply network association analysis to construct an association network diagram between multi-omics features and clinical phenotypes. Finally, Cytoscape software will be used to visualise the generated network diagram, intuitively displaying the complex relationship between each omics feature and clinical phenotype.

#### 2.4.8 Data management and security protocols

Study data will be collected by the researchers and recorded in a standardised medical record report form. The names of participating patients will be replaced with ID numbers and medical record numbers to protect patient privacy. Data entry will be performed using an electronic data collection (EDC) system (http://121.229.17.89:9080/NewXYEDC/), which is constructed by the National Drug Clinical Trial Facility (GCP Centre) of Xiyuan Hospital, China Academy of Chinese Medical Sciences. After data collection and entry are completed, the GCP Centre staff and the Principal Investigator will conduct data review and cleaning to ensure data completeness and accuracy.

## 3 Discussion

Cardiovascular diseases and associated vascular risk factors exhibit a robust correlation with cognitive decline. Among patients diagnosed with stable CHD, cognitive impairment was documented at an incidence of 42.4% following a 3.7-year follow-up period (15). This risk escalates with advancing age, as evidenced by the prevalence of MCI reaching 15.5% among individuals aged over 60 years in China (16). Consequently, there is a pressing need for increased research focus on cognitive function in elderly patients with CHD. A prior investigation revealed that the prevalence of MCI in CHD patients exceeding 55 years of age has attained 55% (10). Nevertheless, the exact prevalence of MCI in the elderly population over 65 years of age requires further elucidation through additional research. Additionally, the development of early diagnostic prevention strategies and the mitigation of MCI progression to enhance the quality of life in the elderly population constitute critical research priorities.

Regarding cognitive function assessment, the diagnostic efficacy and predictive capacity for cognitive impairment risk using neuropsychological scales alone remain insufficiently substantiated. Recent studies have demonstrated superior predictive outcomes for MCI risk in CHD patients when incorporating clinical data analysis (10). The advent of high-throughput technologies has facilitated the integration of multi-omics data with phenomics data, enabling the identification of differential molecular expression patterns. This approach offers a comprehensive framework for characterisation across both macroscopic and microscopic dimensions, potentially enhancing the prediction of CHD-associated MCI in elderly populations.

In this investigation, we will collect comprehensive clinical data combined with multi-omics analyses, including blood metabolomics, proteomics, metagenomics, and neuroimaging data. Using high-throughput technologies, we will conduct comparative analyses of metabolic profiles, protein expression, microbiota composition, and neuroimaging parameters between different patient cohorts. We will establish correlations between micro-phenotypic features (multi-omics data and laboratory indices) and macro-phenotypic clinical manifestations (physiological and pathological characteristics), and will construct a multi-omics network map to elucidate the potential mechanisms underlying MCI in elderly patients with CHD.

Through the synthesis of multi-omics data, this study facilitates a more comprehensive analysis of clinical and molecular characteristics in elderly patients with CHD and concomitant MCI, thereby enhancing predictive capabilities for disease risk assessment and informing early intervention strategies. However, due to temporal constraints, this cross-sectional study design will preclude the determination of the temporal relationship between CHD and cognitive impairment onset in elderly patients, nor can it ascertain the precise incidence rates and temporal patterns of cognitive impairment in this population. These limitations underscore the necessity for more extensive prospective studies and longitudinal follow-up to obtain more accurate epidemiological data. Furthermore, building upon these multi-omics findings, future experimental investigations will be conducted to elucidate the intrinsic mechanisms underlying MCI development in elderly CHD patients, thereby informing clinical prevention and treatment strategies.
